# Apical fibrobullous lung disease in spondyloarthritis patients with biologic DMARDs indication

**DOI:** 10.1093/rheumatology/kead361

**Published:** 2023-07-27

**Authors:** Zehra Ozsoy, Gizem Ayan, Gamze Durhan, Umut Kalyoncu

**Affiliations:** Faculty of Medicine, Department of Internal Medicine, Division of Rheumatology, Hacettepe University, Ankara, Turkey; Faculty of Medicine, Department of Internal Medicine, Division of Rheumatology, Hacettepe University, Ankara, Turkey; Faculty of Medicine, Department of Radiology, Hacettepe University, Ankara, Turkey; Faculty of Medicine, Department of Internal Medicine, Division of Rheumatology, Hacettepe University, Ankara, Turkey

**Keywords:** spondyloarthropathies, apical fibrobullous change, bDMARDs, QuantiFERON-TB gold plus

## Abstract

**Objective:**

The rate of pleuroparenchymal involvement in patients with SpA varies widely, from 0% to 85%. The most common form is apical fibrobullous disease (AFLD). The aim of this study was to determine the incidence of AFLD and associated factors in SpA patients under and/or planned to start biologic DMARDs (bDMARDs) therapy.

**Methods:**

The records of 3021 SPA patients registered with HUR-BIO who had indication of bDMARDs between 2010 and 2021 were scanned. The study included 2489 patients with at least one chest radiograph (X-ray). Patient demographics, comorbidities, laboratory data, bDMARDs used, baseline DASs, and purified protein derivative and/or QuantiFERON test results before initiation of bDMARDs were recorded.

**Results:**

Of the 2489 patients, 36 (1.4%) were found to have AFLD by X-ray and/or CT. The mean disease duration was 11.7 (7.1) years. Patients with AFLD were more likely to be male [28 (77.8%) *vs* 1321 (53.9%), *P* = 0.004], older [56.3 (10.5) years *vs* 44.8 (11.4) years, *P* < 0.001], heavy smokers [27 (79.4%) *vs* 1468 (60.9%), *P* = 0.028] and have had longer disease duration [17. 7 (9.7) years *vs* 11.6 (7) years, *P* = 0.001]. QuantiFERON positivity was higher in the AFLD group [9 (36%) *vs* 309 (16.1%), *P* = 0.013]. While treatment with adalimumab was less preferred in those with AFLD, treatment with etanercept was more frequently preferred.

**Conclusion:**

As the radiological findings of AFLD can be confused with those of tuberculosis, special attention should be paid to differentiating between tuberculosis and the disease in males and in patients who have had long disease duration.

Rheumatology key messages

- Apical fibrobullous lung disease in SpA patients is not very common.
- A serious involvement can be seen in males and in those with advanced disease duration.
- In this patient group, planned to start b-DMARDs, more care should be taken to differentiate TB from apical fibrobullous lung disease.

## Introduction

SpAs are a group of multisystem inflammatory seronegative arthritis diseases characterized by inflammation of the vertebra, peripheral joints and periarticular structures. SpA includes AS, ReA, PsA and enteropathic arthritis. Common characteristic features of SpA are spinal and peripheral arthritis involvement characterized by synovitis and enthesitis [1]. Extra-articular findings include ocular, cardiac, renal, dermatological, gastrointestinal, and pulmonary involvement. Pulmonary involvement (as extra-articular manifestation) is often underestimated by clinicians due to its very low incidence. A large study of 2080 SpA patients reported 1.3% pleuropulmonary involvement [2]. X-ray or high-resolution CT (HRCT) studies have reported rates of pleuroparenchymal involvement ranging from 0% to 85% [3]. The forms of pleuroparenchymal involvement are upper lobe fibrobullous disease, interstitial lung disease, pleural thickening, pleural effusion, and mycetoma [4]. The most common form of pulmonary involvement in SpAs is apical fibrobullous lung disease (AFLD). It presents with bilateral fibrosis with apical pleural thickening, which characteristically begins as patchy consolidation at both apexes. Histopathological examination of lung biopsies reveals non-specific chronic inflammation and fibrosis. Although prevalence data vary, it has been reported to range from 1.3% to 30%. It is more common in men and is usually unilateral in the early stages and bilateral in the long term. In addition, when SpA duration is over 15 years, the likelihood of developing AFLD increases. AFLD is usually asymptomatic unless it covers a very large area or if there are secondary infections. In advanced cases, cough, sputum production, and dyspnoea may develop [5]. Biologic DMARDs (bDMARDs) have been used successfully in active, difficult-to-control SPA patients [6]. Tuberculosis (TB) screening is performed in patients with SpA prior to starting bDMARDs treatment. The most commonly used tests for TB screening are the purified protein derivative (PPD) and/or Quantiferon (QFT) tests. In addition, all patients should have an anteroposterior chest X-ray before treatment; if there is evidence of previous TB, TB prophylaxis (often isoniazid) should be started before bDMARDs treatment. Apical changes in the chest X-ray are one of the most common findings in TB. Fibrotic changes in this region in patients with SpA complicate the differential diagnosis. Therefore, it may be difficult to exclude TB in the presence of apical fibrobullous changes in patients receiving bDMARDs [7].

The aims of this study were (a) to determine the frequency of apical fibrobullous changes on chest X-ray and/or thorax CT in patients with SpA who are receiving and/or for whom it is planned that they receive bDMARDs therapy; (b) to compare the characteristics of the patient groups with and without apical fibrobullous changes.

## Method

### Patient selection

HUR-BIO is a single-centre database established in 2005 to register and follow patients with rheumatological diseases who will be started on bDMARDs treatment [8]. The files and records of 3021 patients registered in HUR-BIO who were diagnosed with SpA and for whom it was planned that they receive bDMARDs between 2010 and 2021 were scanned. This was a single-centre, retrospective, descriptive study without a control group. The SpA disease group included AS patients (according to the modified New York criteria), non-radiographic axial SpA (nrAxSpA) patients (according to the Assessment of SpondyloArthritis international Society criteria), axial PsA and/or at least one syndesmophyte detected in the lumbar spine.

### A definition of lung apical fibrobullous changes

Of the 3021 patients with a diagnosis of SpA registered in HUR-BIO, 2489 patients with a chest X-ray recorded at least once in the Picture Archiving Communication Systems (PACS) were included in the study. The chest X-ray was assessed by two different rheumatologists and one radiologist. The evaluating rheumatologists and the radiologist were not informed about the clinical findings for the patients in order to prevent possible bias in their interpretations. Tuberculosis was excluded if the patient did not have lung CT and there was no calcification according to the chest X-ray.

**Figure 1.**
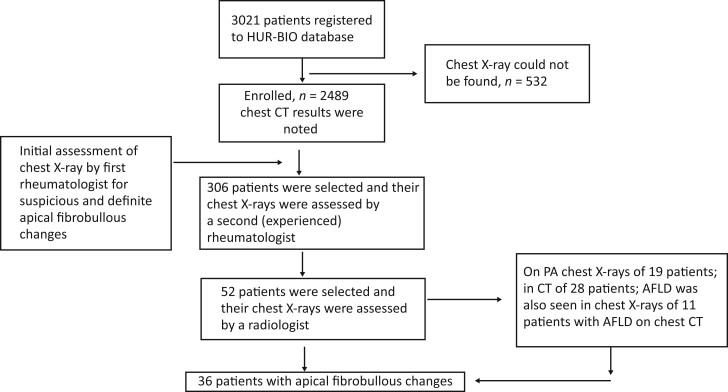
Flow chart of patient selection procedure. AFLD: apical fibrobullous disease; PA: posterior anterior

After the initial assessment of the chest X-ray by the rheumatologist, 306 patients for whom AFLD was strongly suspected were selected. The re-evaluation of these 306 patients by an experienced rheumatologist identified 52 patients with definite findings. The radiological images of these patients were re-evaluated by an experienced radiologist. Apical fibrosis was considered present if the apical diameter measured at the first subcostal margin in the chest X-ray was 5 mm or more. Apical changes were also noted in 241 patients with thorax CT images included in the PACS. In all, 36 patients were found to have apical fibrobullous changes on chest X-ray and/or thorax CT (Fig. 1).

### Study parameters

Demographic characteristics included data such as age, sex, date of birth, marital status, educational status, date of diagnosis, disease duration, chronic obstructive pulmonary disease and other pulmonary comorbidities (e.g. asthma), other comorbidities (e.g. diabetes mellitus, hypertension, chronic renal failure, coronary artery disease, malignancy), smoking, duration of smoking if used, BMI, extra-articular findings (psoriasis, uveitis, IBD), HLA-B27 results, ESR, CRP levels, bDMARDs used (anti-TNF drugs such as infliximab, adalimumab, etanercept, certolizumab, golimumab, IL-17 blockers such as secukinumab, ustekunimumab, etc.), cDMARDs (MTX, LEF, SSZ) used, steroid doses, and baseline disease activity [BASDAI, BASFI, visual analogue score (VAS) Pain, VAS Fatigue, VAS Global, HAQ] of SpA patients enrolled in the study. In addition, the result of the PPD and/or QFT test, which was routinely performed prior to starting bDMARDs, was recorded. QFT test results were classified as negative or positive. PPD results were divided into three classes: 4 mm and below, 5–10 mm, and 11 mm and above.

In patients with SpA, the frequency of apical fibrosis was determined by chest X-ray and/or CT. Demographic data for patients with and without apical fibrosis (including disease duration, comorbidities, smoking status, BMI, extra-articular findings, HLA-B27 results, ESR, CRP and hemogram values, baseline disease activity, bDMARD selections, QFT, and PPD values) were compared.

### QuantiFERON-TB gold plus

QFT-Plus is an ELISA-based test that measures the IFN-γ response of T cells stimulated by *Mycobacterium* TB antigens (ESAT-6 and CFP-10); the TB1 tube contains the ESAT-6 and CFP-10 peptide antigens that induce a CD4+ T cell response, while the TB2 tube contains additional shorter peptides from ESAT-6 and CFP-10, designed to stimulate both CD4+ and CD8+ T cells [9]. The QFT-Plus test was performed and evaluated according to the manufacturer’s recommendations [10]. QFT plus test results were recorded as positive, negative or indeterminate.

### Tuberculin skin test

After a PPD administered by intradermal injection, called the Mantoux method, the largest induration diameter was reported; 5 mm and above was considered positive due to partial immunosuppression in inflammatory rheumatological patients.

### Ethical consideration

Our study was conducted in accordance with the 2013 amendment of the Declaration of Helsinki, ethical approval was obtained from Hacettepe University Institutional Review Board (GO-21/914) and written informed consent for participation was obtained from each participant.

### Statistical analysis

Statistical analysis was performed using SPSS Statistics for Windows, version 23.0 (IBM SPSS Statistics for Windows, version 23.0. Armonk, NY: IBM Corp). The normality of numerical variables was tested by visual (histogram and probability plots) and analytical methods (Kolmogorov–Smirnov and Shapiro–Wilk tests). Descriptive analyses were presented as mean ± S.D. for continuous variables and as frequencies and percentages for categorical variables. In independent groups, categorical data and rates were compared among groups using the eligibility criteria, χ^2^ or Fisher tests. The independent samples *t* test compared the means of two independent groups for normally distributed parameters. *P* < 0.05 was accepted as statistically significant.

## Results

### Demographic findings

The study included 2489 patients (Fig. 1). Of these, 1349 (54.2%) patients were male, and the mean age was 45.1 (11.5) years. The mean disease duration was 11.7 (7.1) years. Regarding the educational level, 1679 (68.5%) patients had a high-school degree or higher, and 773 (31.5%) patients had less than a high-school degree.

### Frequency and distribution of apical fibrosis in patients with SpA

AFLD was detected in 36/2489 (1.4%) patients. The frequency of AFLD in patients with disease duration of <5 years, 5–10 years, >10 years and >20 years was as follows: 1 (0.3%), 7 (0.8%), 15 (1.4%), 13 (4.3%); (*P* ≤ 0.001). The incidence of AFLD was 8 (0.70%) in women and 28 (2.1%) in men; (*P* = 0.004). The results of the analysis of the frequency of apical fibrosis according to gender and disease duration are shown in Table 1. Lung CT was available in 241 of 2489 patients. AFLD was found in 19 (0.76%) patients on chest X-ray and in 28 (11.6%) patients on CT. Apical fibrosis was present in the lung CT of 17 patients who were chest X-ray negative. In 11 patients (39.3%) with AFLD on CT, AFLD was also seen on chest X-ray. However, in our study, the changes in 4 patients with QFT positivity and no CT, but apical fibrobullous changes in PAC, were attributed to rheumatologic disease because they did not contain calcifications, which can be seen more frequently in TB. According to chest X-ray, 5 (0.2%) patients had right-side AFLD, 8 (0.32%) patients had left-side AFLD, and 9 (0.36%) patients had bilateral AFLD.

**Table 1.** Frequency according to gender and duration of disease

Duration of disease	Male *n* (%)	Female *n* (%)	Total *n* (%)
>20 years	12 (5.7)	1 (1.1)	13 (4.3)
>10 years	11 (1.9)	4 (0.8)	15 (1.4)
5–10 years	4 (0.9)	3 (0.8)	7 (0.8)
<5 years	1 (0.7)	0 (0)	1 (0.3)
Total	28 (2.1)	8 (0.7)	36 (1.4)

### Characteristics of patients with and without apical fibrosis in SpA

The distribution of apical fibrobullous change according to age (in decades) is shown in Table 2. Patients with AFLD were more likely to be male [28 (77.8%) *vs* 1321 (53.9%), *P* = 0.004], be older [56.3 (10.5) years *vs* 44.8 (11.4) years, *P* ≤ 0.001], have longer disease duration [17. 7 (9.7) years *vs* 11.6 (7) years, *P* = 0.001)], have lower BMI [25.6 (3.2) *vs* 27.8 (5.5), *P* ≤ 0.001] and be heavy smokers [27 (79.4%) *vs* 1468 (60.9%), *P* = 0.028] (Table 3). Twenty-two (64.7%) of the patients in the group with AFLD and 1657 (68.5%) of the patients in the group without AFLD had high-school education or higher (*P* = 0.63). The frequency of uveitis [4 (12.1%) *vs* 272 (11.3%), *P* = 0.78], psoriasis [1 (2.9%) *vs* 37 (1.5%), *P* = 0.41] and IBD [1 (3%) *vs* 106 (4.4%), *P* = 1.00] was similar in patients with and without AFLD. Overall, HLA-B27 was positive in 708 (47%) patients and negative in 797 (53%) patients. HLA-B27 was positive in 5 (38.5%) and negative in 8 (61.5%) patients with AFLD. There was no statistically significant difference in HLA-B27 positivity (*P* = 0.534). Patients' disease activities and functions were similar at initiation of bDMARDs (Table 3). Asthma [0 (0%) *vs* 100 (6.1%), *P* = 0.63] and COPD [1 (4.8%) *vs* 6 (0.4%), *P* = 0.085] were similar in patients with and without AFLD.

**Table 2.** Comparison of age (in decades) in patients with and without apical fibrobullous changes in the lung

Age, decades	Apical fibrobullous changes *n* (%)	No apical fibrobullous changes *n* (%)	Total *n* (%)
20–29	0 (0)	185 (7.5)	185 (7.4)
30–39	3 (8.3)	687 (28)	690 (27.7)
40–49	5 (13.9)	774 (31.6)	779 (31.3)
50–59	14 (38.9)	524 (21.4)	538 (21.6)
60–69	12 (33.3)	234 (9.5)	246 (9.9)
70–80	2 (5.6)	49 (2)	51 (2)
Total	2453	36	2489

**Table 3.** Comparison of the SPA patient groups with apical fibrobullous changes in the lungs and those without apical fibrobullous changes in the lungs

		Apical fibrobullous changes *n* (%): 36 (1.4)	No apical fibrobullous changes *n* (%): 2453 (98.6)	*P*-value
Gender, male, *n* (%)		28 (77.8)	1321 (53.9)	0.004
Age, mean (s.d.)		56.3 (10.5)	44.8 (11.4)	<0.001
Disease duration, mean (s.d.)		17.7 (9.7)	11.6 (7)	0.001
Disease duration, *n* (%)	<5 years	1 (2.8)	301 (12.3)	<0.001^a^
	5–10 years	7 (19.4)	818 (33.3)	
	10 years and above	15 (41.7)	1046 (42.6)	
	20 years and above	13 (36.1)	288 (11.7)	
BMI, mean (s.d.)		25.6 (3.2)	27.8 (5.5)	<0.001
Smoking, *N* (%)	Never	7 (20.6)	944 (39.1)	0.028 (ever *vs* never)
	Current	17 (50)	1009 (41.8)	
	Ex	10 (29.4)	459 (19)	
PPD, *n* (%)	<4 mm	3 (23.1)	121 (21.1)	1.00
	5-10 mm	3 (23.1)	154 (26.9)	
	Above 11 mm	7 (53.8)	298 (52)	
QFT test positive, *n* (%)		9/25 (36.0)	309/1925 (16.1)	0.013
Sedimentation, mm/h, mean (s.d.)		30.3 (22.4)	25.8 (22.2)	0.269
CRP, mg/dl, mean (s.d.)		2.7 (2.6)	2.4 (4.5)	0.756
Outcome measures	BASDAI, mean (s.d.), (0-100)	52.2 (17)	57.3 (20.5)	0.211
	BASFI, mean (s.d.), (0–100)	43.2 (26)	44.8 (25.2)	0.766
	HAQ, mean (s.d.), (0-3)	0.3 (0.2)	0.6 (0.5)	0.22
	VAS Global, mean (s.d.), (0–100)	52.6 (22.5)	62.8 (28.6)	0.089
	VAS Pain, mean (s.d.), (0–100)	62.9 (17.6)	66.7 (22.6)	0.462
	VAS Fatigue, mean (s.d.), (0–100)	55.3 (22.4)	57.7 (28)	0.704

a Disease duration was found to differ between the four groups (*P* < 0.001). When the groups were compared in pairs, it was seen that the difference was between the patient groups for <5 years and for 20 years and more (*P* < 0.001), the patient groups for 5–10 years and for 20 years or more (*P* < 0.001), and the patient groups for 10–20 years and for 20 years or more (*P* < 0.001). There was no difference between the <5 years and 5–10 years patient groups (*P* = 0.69), the <5 years and 10–20 years patient groups (*P* = 0.21), or the 5–10 years and 10–20 years patient groups (*P* = 0.38). PPD: purified protein derivative; QFT: Quantiferon test; VAS: visual analogue scale.

QFT positivity was detected in 318 (16.3%) patients. While QFT positivity was higher in the group with apical fibrobullous changes [9 (36%) *vs* 309 (16.1%), *P* = 0.013], no difference was found between the PPD results. (Table 3). The use of conventional synthetic DMARDs (csDMARDs), first bDMARDs and ever bDMARDs in patients with AFLD is shown in Table 3. While adalimumab is less preferred in patients with AFLD, etanercept seems to be more preferred (Table 4).

**Table 4.** Comparison of first b-DMARDS, ever b-DMARDS, and previous cs-DMARDS in patients with and without apical fibrobullous changes in the lung

		Apical fibrobullous changes in the lung *n* (%)	No apical fibrobullous changes in the lung *n* (%)	*P*-value
First b-DMARDS	Adalimumab, *n* (%)	9 (26.5)	942 (38.8)	0.144
	Etanercept, *n* (%)	10 (29.4)	533 (21.9)	0.296
	Infliximab, *n* (%)	12 (35.3)	417 (17.2)	0.006
	Golimumab, *n* (%)	2 (5.9)	261 (10.7)	0.574
	Certolizumab, *n* (%)	1 (2.9)	249 (10.2)	0.25
	Secukinumab, *n* (%)	0 (0)	28 (1.2)	1.00
Ever b-DMARDS	Adalimumab, *n* (%)	11 (30.6)	1381 (56.3)	0.002
	Etanercept, *n* (%)	20 (55.6)	924 (37.7)	0.028
	Infliximab, *n* (%)	16 (44.4)	589 (24)	0.005
	Golimumab, *n* (%)	6 (16.7)	397 (16.2)	0.938
	Certolizumab, *n* (%)	4 (11.1)	620 (25.3)	0.052
	Secukinumab, *n* (%)	4 (11.1)	240 (9.8)	0.775
	Ixekizumab, n (%)	0 (0)	5 (0.2)	1.00
Previous cs-DMARDS	MTX, *n* (%)	16 (47.1)	728 (30.1)	0.033
	LEF, *n* (%)	3 (8.8)	158 (6.5)	0.486
	SSZ, *n* (%)	30 (88.2)	1804 (74.7)	0.07
	Glucocorticoids, *n* (%)	14 (41.2)	619 (25.6)	0.04

b-DMARDS: biologic DMARDS; cs-DMARD: conventional synthetic DMARD.

## Discussion

In this study, the incidence of AFLD on chest X-ray and/or thorax CT was found to be 1.4% in SpA patients using bDMARDs. Compared with previous studies, our study had the highest number of patients, and radiological images were interpreted by two rheumatologists and a radiologist. When the studies in the literature are examined, in the majority of articles, it is reported that radiological images were evaluated only by a single radiologist.

The incidence of AFLD increased with age, particularly in men. While smoking was more common in patients with AFLD, the incidence of latent TB was higher than that of smoking. Some bDMARDs (such as etanercept) were more commonly preferred in this patient group.

Many studies have been published on the incidence of AFLD in SpA. In earlier studies, Hunninghake and Rumancik found that the frequency of AFLD varied over a wide range between 1.3% and 30% [11, 12]. In one of the largest series in the literature, Rosenow from the Mayo Clinic reviewed chest X-rays of 2080 patients with AS and found apical fibrosis in 26 patients (1.2%) [2]. This rate is consistent with the 1.4% we found. As not all of the patients in our study had thorax scans, it would be expected that the frequency of AFLD we detected would be at the lowest rate. However, it can be said that there are no findings compatible with the apparent AFLD on chest X-ray.

The two most important risk factors for developing AFLD in patients with SpA are disease duration and gender. The mean time from the common signs of the disease to the development of AFLD was 11.7 (7.1) years. Rumancik and Gupta stated that this period varies between 6 and 35 years [12, 13]. Our study also supports these findings. Indeed, in our study, while the frequency of AFLD was 0.3% in SpA patients with a disease duration of less than 5 years, and 0.8% for those with a disease duration of between 5 and 10 years, this frequency increased to 1.4% in patients with a disease duration of over 10 years and to 4.3% with a disease duration of over 20 years. The male gender is associated with more severe disease involvement in SpA patients. They have a radiographically advanced level of disease and have worse functional status [14]. Similarly, the disease occurs more frequently, especially in male patients, in patients with a disease duration of >20 years. This suggests that AFLD may be associated with advanced disease. However, important limitations of our study: We could not differentiate between axial SpA (rAxSpA) and nr-AxSpA in the diagnosis of the patients, we did not have records of Scoring radiographic progression in ankylosing spondylitis (mSASSS) or syndesmophyte counts that would indicate radiologic progression of the patients, and we did not have records of BASDAI and ASDAS follow-up values, which are the disease activity indexes of the patients.

Rosenow published the claim that the most common pulmonary pathology detected in patients with AS is AFLD [2]. There are two main theories of pleuropulmonary involvement in SpA patients: mechanical and disease-specific. Based on the mechanical theory, hyperventilation at the base of the lung in SpA patients is blamed for the ventilatory dysfunction caused by hypoventilation at the apex. The fact that the disease is more common with axial joint involvement and less common with peripheral joint involvement supports the mechanical theory [15, 16]. The other disease-specific theory is based on Campbell and MacDonald’s case report of the development of apical fibrosis 2 years before the onset of joint symptoms [17, 18]. In the Bouvier study, no statistically significant difference was found between the upper and lower lobes in terms of mucociliary activity between the control group and the patients with apical fibrosis. Therefore, they concluded that AFLD develops by a specific mechanism related to the nature of SpA [19].

Our patient group consisted of SpA patients using bDMARDs. In these patients, latent TB screening is performed before commencement of bDMARDs. The most-used method in latent TB screening is the chest X-ray and PPD/QFT test. Apical fibrosis detected in chest X-ray is particularly confusing, as it makes the clinician consider the possibility of TB. QFT positivity was found much more frequently in our patients with AFLD than in those without (36.0% *vs* 16.0%). Interestingly, in a study by Ho H *et al.* evaluating 2136 AS patients, AFLD was found in 63 patients (2.9%). It was observed that secondary fibrosis developed as a result of TB in 40 of these patients, and as a result of inflammation of AS in 22 [20]. This study suggests that TB may also play a role in the development of apical fibrosis in SpA. A systematic review including 88 studies from 36 countries showed the global prevalence of latent tuberculosis infection was 24.8% (95% CI: 19.7–30.0%) [21]. Moreover, in this study, regional and global estimates of latent tuberculosis infection prevalence were calculated and extrapolated to the countries with no prevalence data, including Turkey, using incidence information. It has been stated that the estimated latent TB frequency in Turkey coincides with the intermediate level, which means ∼19% according to the QFT test. In our study, QFT-Plus positivity was found to be 16% in the group without apical fibrobullous changes and was similar to the extrapolated prevalence of general population. However, QFT-Plus positivity was found to be 36% in the group with apical fibrobullous changes, indicating an increased frequency in this group. This suggests that apical fibrobullous changes can be attributed to SpA. Even though these results are convincing, further studies are still needed on this subject, and TB testing should be given emphasis in patients with AFLD. The fact that etanercept was an important treatment option in this patient group in our study suggests that clinicians consider AFLD findings in their treatment selection, considering the lower risk of TB.

Our study showed the differences between patients with AFLD and patients with SpA in a large cohort, including differences in radiological and rheumatological assessments.

The limitations of our study are that it was retrospective, and the detailed TB history of these patients and how many of them had active TB infection at follow-up were not known. In our study, we reported the frequency of apical fibrobullous changes in SpA patients. It is clear that these changes may be due to possible disease activity or to possible TB infection, and this was one of the limitations of our study. Another important limitation of our study was the lack of radiological severity scores for the patients. The chest expansion values and BASMI of only 290 of the 2489 patients included could be obtained. Due to missing data, this measurement could not be evaluated. Therefore, the relationship between AFLD and the radiological score, and therefore the relationship with the severity of the disease, could not be investigated. In addition, in our database there was no information about whether the patients with AFLD developed clinically relevant pulmonary symptoms throughout the follow-up. In our study no relationship was found between HLA-B27 positivity and AFLD. However, HLA-B27 should be re-evaluated in larger cohort studies.

In conclusion, AFLD can be seen in SpA patients, although it is not very common. The increased incidence in males and in those with advanced disease duration suggests that there may be a serious involvement. Findings of AFLD that can be detected with chest X-ray prior to starting bDMARDs treatment may confuse the clinician, so determining whether the current situation is related to TB or disease is an important issue. Given that the incidence of latent TB is higher in this patient group, more care should be taken to differentiate TB from disease, especially in men and patients with long disease duration.

## Data Availability

Data are available upon reasonable request to the corresponding author.
